# Syphilitic Retinitis: A Rare Presentation of a Resurgent Sexually Transmitted Infection in an HIV-Negative Individual

**DOI:** 10.7759/cureus.73273

**Published:** 2024-11-08

**Authors:** Mayuri Phatak, Devendra Venkatramani, Shruti Choudhari, Hetal Shah, Tanvi Haldipurkar, Maninder S Setia

**Affiliations:** 1 Ophthalmology, Laxmi Charitable Trust Eye Hospital, Mumbai, IND; 2 Epidemiology, Laxmi Charitable Trust Eye Hospital, Mumbai, IND

**Keywords:** high clinical suspicion, hiv-negative patients, ocular manifestations, syphilis, syphilitic retinitis

## Abstract

We present a rare presentation of isolated syphilitic retinitis in an HIV-negative man. A 47-year-old male presented to our ophthalmology center with complaints of blurred vision, pain, and redness in the left eye for the past seven days. The best corrected visual acuity for distance was 6/6 and best corrected near visual acuity for near was N6 in the right eye. The best corrected visual acuity for distance was finger counting at 1 m and best corrected near visual acuity for near was <N48 in the left eye. The right eye developed similar features of retinitis after one week; the vision worsened and the best corrected visual acuity for distance was 6/18P and best corrected visual acuity for near was N18. The vision was hand movement and counting fingers in the left eye on this visit. The left eye showed keratic precipitates on the endothelial surface; they were non-granulomatous keratic precipitates. The fundus evaluation with an indirect ophthalmoscope showed dense vitritis with snowballing and yellow colored confluent placoid wreath-like lesions suggestive of acute necrotizing retinitis. The venereal disease research laboratory (VDRL) test was reactive (>1:32), the Treponema Pallidum Hemagglutination Assay was positive, and the patient tested negative for human immunodeficiency virus antibodies. Based on these findings, a diagnosis of syphilitic retinitis was made. The patient was given three doses of 2.4 million units of benzathine penicillin intramuscularly (once a week) and doxycycline 100 mg twice daily for the same period. After completion of treatment, the best corrected visual acuity for distance improved to 6/9 and the best corrected near visual acuity for near improved to N6 in the right eye, and the lesions in the eye resolved. The best corrected visual acuity for distance improved to 6/12 and the best corrected near visual acuity for near improved to N10 in the left eye. If a patient presents with unexplained ophthalmic findings such as uveitis, vitritis, or retinitis, then a diagnosis of syphilis should be considered even if the patient does not give a history of high-risk sexual behaviour. Thus, both the physician at the sexually transmitted infection clinic and the ophthalmologist should be aware of these symptoms and signs and consider this as a potential diagnosis. This will result in prompt investigations, appropriate diagnosis, and clinical management, and eventually prevent loss of vision.

## Introduction

Syphilis, also known as the great imitator or masquerader, is known to affect many organs of the body during various stages of the infection (primary, secondary, latent, and tertiary) [1]. The epidemiology of syphilis has changed over the past two decades. Though there was a reduction in bacterial sexually transmitted infections (STIs), including syphilis, in the 2000s, recent reports have suggested a resurgence of this infection, particularly in high-risk groups such as men who have sex with men [2, 3]. A large epidemiological study found that 1.5% of patients with syphilis had ocular manifestations [4]. We present a rare presentation of isolated syphilitic retinitis in an HIV-negative man.

## Case presentation

**Figure 1:**
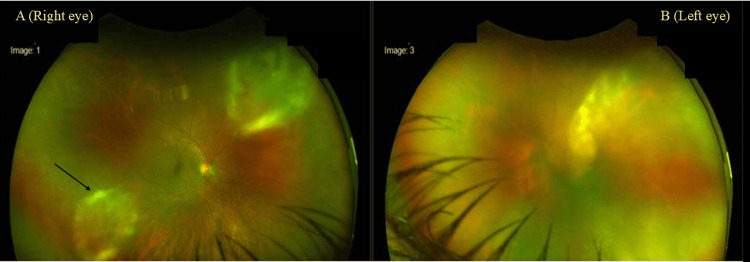
Optos wide-field image showing yellow-colored confluent placoid wreath-like lesions suggestive of retinitis in the right eye (A) and left eye (B).

A 47-year-old male presented to our ophthalmology center with complaints of blurred vision, pain, and redness in the left eye for the past week. Initially, the best corrected visual acuity for distance was 6/6 (Snellen’s chart) and best corrected visual acuity for near was N6 (on the near vision N notation chart) in the right eye. However, the best corrected visual acuity for distance was finger counting at 1 m, and best corrected visual acuity for near was < N48 in the left eye. After one week, the right eye developed similar features. There was a drop in the best corrected visual acuity for distance to 6/18P, and best corrected visual acuity for near was N18 in the right eye, with the vision in the left eye reduced to hand movement and counting fingers on this visit. There were no external lesions on either eye. On a slit lamp examination, the left eye showed non-granulomatous keratic precipitates on the endothelial surface. There were occasional cells and no flare. Fundus evaluation with an indirect ophthalmoscope showed dense vitritis with snowballing and yellow-colored confluent placoid wreath-like lesions suggestive of acute necrotizing retinitis. As indicated earlier, the right eye developed similar features of retinitis after one week. An image taken on an Optos wide field fundus camera (Optos®) showed placoid wreath-like lesions (Figure 1). The right eye had two retinitis lesions with a wreath-like pattern, superonasally and inferotemporally. The left eye showed necrotizing retinitis inferiorly and temporally; however, due to dense vitritis, details were not visible.

**Figure 2:**
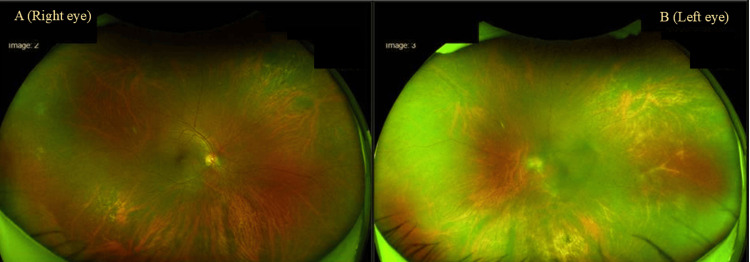
Image showing resolved lesions in the right eye (A) and the left eye (B).

Optical coherence tomography was not performed as the lesions did not involve the macula, and no macular edema was clinically found. There were no other lesions on the body, and the patient did not report any history of lesions or ulcers on any part of the body. There were no other systemic complaints and no signs or symptoms of other nerve dysfunction. Based on these features, a differential diagnosis of infective retinitis (such as viral or bacterial retinitis) was made. The Mantoux test was negative and radiological examination of the chest was normal. The blood and urine cultures did not grow any organisms. The complete blood count and erythrocyte sedimentation rate were within normal limits. Thus, other infective causes were ruled out. However, the venereal disease research laboratory (VDRL) test was reactive (>1:32), the Treponema Pallidum Hemagglutination Assay (TPHA) test was positive (no titres mentioned), and the patient was negative for HIV antibodies. The TORCH tests were done, and they were negative. Based on these clinical and laboratory findings, a diagnosis of syphilitic retinitis was made. The patient was given three doses of 2.4 million units of benzathine penicillin intramuscularly (once a week) along with doxycycline 100 mg twice daily for the same period. After completion of treatment, the best corrected visual acuity for distance improved to 6/9 and best corrected visual acuity for near improved to N6 in the right eye, and the lesions in the eye resolved (Figure 2). The best corrected visual acuity for distance improved to 6/12 and best corrected visual acuity for near improved to N10 in the left eye. Despite repeated attempts, the patient refused to follow up for eye examination and blood tests.

## Discussion

There have been numerous reports of an increase in syphilis cases among men who have sex with men (MSM), individuals who report high-risk sexual behavior, individuals with blood transfusions, and those infected with HIV [2, 5-9]. There has also been an increase in the reporting of ocular syphilis cases, particularly in underserved areas [5, 7]. Ocular manifestations are seen in about 0.6-2% of all patients with syphilis and may occur at any stage of the disease [10]. The patient can present with varied clinical manifestations; the most common manifestation in HIV-negative patients is posterior uveitis [10-12]. However, the common diagnosis in HIV-positive patients is pan-uveitis [12]. Syphilitic retinitis progresses slowly, and placoid confluent lesions are seen in this condition, generally healing without disrupting the underlying retinal pigment epithelium [13, 14]. Necrotizing retinitis, a rare but serious complication of ocular syphilis (mottled appearance), may be similar to acute retinal necrosis (homogeneous) [10, 12]. It shows a robust response to intravenous penicillin. Early antibiotic treatment, the absence of HIV coinfection/neurosyphilis, and better visual acuity at presentation are predictive of achieving good visual acuity at the end of treatment [15].

Tam EK et al. reported the resurgence of ocular syphilis in the United States [7]. They found that 17.5% of individuals who were syphilis-positive had ocular syphilis. The common presentations were anterior uveitis and posterior segment involvement, while the rare presentations included vasculitis, retinal detachment, and neovascular glaucoma [7]. Ahmed and coworkers compared the ocular lesions of syphilis in HIV-negative and -positive individuals [16]. They reported that panuveitis was common in both HIV-negative and -positive patients, whereas diffuse necrotizing retinitis was more common in HIV-infected individuals [16]. Xu Y et al. reported that vision loss, as seen in our patient, could be an indicator of ocular syphilis [17]. Jiang Z et al. suggested that ultra-wide imaging may be useful in diagnosing ocular syphilis [18]. Though some previous case reports have described necrotizing retinitis in HIV-infected individuals, our case was in an HIV-negative patient [19, 20].

It may be difficult to diagnose syphilitic retinitis in patients with severe vitritis. Non-treponemal tests such as rapid plasma regain or the VDRL test may be unreliable and must be combined with a treponemal test like the TPHA [10, 11, 14]. The sensitivity of VDRL ranges from 71% to 100% (it is most sensitive for secondary syphilis) and the specificity is 98% (range 96% to 99%) [21]. The sensitivity of TPHA is >95% and the specificity is >99% [22]. Thus, the tests used for diagnosis had high sensitivity and specificity. In our case, we could not elicit high-risk behavior, probably due to the fact that the patient presented in the eye clinic rather than the STI clinic. As stated above, syphilis cases have been on the rise. Since most of these cases will present to Dermatology or STI clinics, dermatologists should be trained to identify rare complications of syphilis. A complete evaluation of a patient presenting with syphilis should include a history of visual problems and a detailed ophthalmologic examination.

## Conclusions

If a patient presents with unexplained ophthalmic findings such as uveitis, vitritis, or retinitis, a diagnosis of syphilis should be considered even if the patient does not report a history of high-risk sexual behavior. Therefore, both physicians at sexually transmitted infection clinics and ophthalmologists should be aware of these symptoms and signs and consider this as a potential diagnosis. This approach will lead to prompt investigations, appropriate diagnosis, and clinical management, ultimately helping to prevent loss of vision.
